# Amyloidogenic determinants are usually not buried

**DOI:** 10.1186/1472-6807-9-44

**Published:** 2009-07-09

**Authors:** Kimon K Frousios, Vassiliki A Iconomidou, Carolina-Maria Karletidi, Stavros J Hamodrakas

**Affiliations:** 1 Department of Cell Biology and Biophysics, Faculty of Biology, University of Athens, Panepistimiopolis, Athens 157 01, Greece

## Abstract

**Background:**

Amyloidoses are a group of usually fatal diseases, probably caused by protein misfolding and subsequent aggregation into amyloid fibrillar deposits. The mechanisms involved in amyloid fibril formation are largely unknown and are the subject of current, intensive research. In an attempt to identify possible amyloidogenic regions in proteins for further experimental investigation, we have developed and present here a publicly available online tool that utilizes five different and independently published methods, to form a consensus prediction of amyloidogenic regions in proteins, using only protein primary structure data.

**Results:**

It appears that the consensus prediction tool is slightly more objective than individual prediction methods alone and suggests several previously not identified amino acid stretches as potential amyloidogenic determinants, which (although several of them may be overpredictions) require further experimental studies. The tool is available at: . Utilizing molecular graphics programs, like O and PyMOL, as well as the algorithm DSSP, it was found that nearly all experimentally verified amyloidogenic determinants (short peptide stretches favouring aggregation and subsequent amyloid formation), and several predicted, with the aid of the tool AMYLPRED, but not experimentally verified amyloidogenic determinants, are located on the surface of the relevant amyloidogenic proteins. This finding may be important in efforts directed towards inhibiting amyloid fibril formation.

**Conclusion:**

The most significant result of this work is the observation that virtually all, to date, experimentally determined amyloidogenic determinants and the majority of predicted, but not yet experimentally verified short amyloidogenic stretches, lie 'exposed' on the surface of the relevant amyloidogenic proteins, and also several of them have the ability to act as conformational 'switches'. Experiments, focused on these fragments, should be performed to test this idea.

## Background

Amyloidoses are diseases that occur when soluble proteins undergo conformational re-arrangements and form fibrillar aggregates known as amyloid deposits. Such diseases include Alzheimer's, Parkinson's, Creutzfeldt-Jacob's and Huntington's neurodegenerative diseases, as well as type II diabetes, prion diseases and many more. Amyloidogenic proteins are quite diverse, with little similarity in sequence and native 3D-structure [1,2]. Additionally, several proteins and peptides not related to amyloidoses have also been shown to have the potential to form amyloid fibrils *in vitro*, suggesting that this ability for structural rearrangement and aggregation may be inherent to proteins [3].

Despite the diversity of origin, all amyloid fibrils share the same cross-beta architecture and several functional proteins found in bacteria, fungi, insects and humans have also been found to adopt the same architecture under physiological conditions, as part of their functional role [[4-8] and references therein], following our proposal for the existence of natural protective amyloids [9,10].

Evidence indicates that short sequence stretches may be responsible for amyloid formation [11,12] and several methods have been published recently, that attempt to predict amyloidogenic regions, based on various properties of proteins [[13] (TANGO), [14-20] (PASTA), [21] (AGGRESCAN), [22,23] (SALSA), [24] (Zyggregator)].

Each method makes its own assumptions and implements its own predictors, which range from quite simplistic to quite complex. The ability to form β-strands is a predominant feature in most works, either in the form of statistical propensities or in the form of structural stability. Yoon and Welsh (2004) searched for hidden beta-propensity in sequences, in other words regions that appear to be natively α-helical but have nonetheless the ability to form β-strands. Hamodrakas et al. [25] have similarly looked for "conformational switches" in sequences -regions with a high predicted tendency to form both α-helices and β-strands- using the consensus secondary structure prediction program SecStr [26] and Zibaee et al. [23] looked for β-contiguity, essentially a derivative of β-strand propensity based on the Chou and Fasman [27,28] set of secondary structure preference values. In a more structural approach, Thompson et al. [19] and Zhang et al. [22] identified regions computationally that can be stable as β-strands in a stacked β-sheet crystal, similar to the one obtained from the peptides GNNQQNY and NNQQNY [29], known amyloidogenic regions from the yeast prion Sup35, while Trovato et al. [20], looked for regions with the ability to pair with each other and form β-sheets, with their program termed "PASTA".

The formation of β-strands is not the only predictor though. Conchillo-Solé et al. [21] defined a set of aggregation propensities, upon which they calculate the presence of aggregation "hot-spots" in sequences. Galzitskaya et al. [17,18] also defined a novel intrinsic property for aminoacid residues, the average expected packing density, which they found to be correlated to amyloidogenesis, while Lopez de la Paz and Serrano [11] identified a sequence pattern that is involved in the formation of amyloid-like fibrils.

A variety of multi-parametric methods exist as well. Pawar et al. [16] and Tartaglia et al. [24] combine intrinsic properties of aminoacid sequences to calculate aggregation propensities, while Tartaglia et al. [24] and Fernandez-Escamilla et al. [13] additionally include the effect of environmental variables in their equations for calculating aggregation rates.

We demonstrated that a consensus approach might be better suited for the task of predicting amyloidogenic stretches [25] and we developed a consensus algorithm, AMYLPRED, described below (freely available for academic users at ), which combines some of the these methods, representing most of the above mentioned categories.

As mentioned above, amyloidogenic proteins are quite diverse, with little similarity in sequence and native 3D-structure [1,2,30]. Therefore, we tried to determine a common molecular denominator to all amyloid fibril favouring regions, the so-called amyloidogenic determinants, which may dictate their ability at molecular level to form amyloid fibrils. Our efforts were guided by the superb work of Sawaya et al. [31], who reported that as many as 30 segments from fibril-forming proteins that form amyloid-fibrils, microcrystals, or usually both, all form dry 'steric zippers', which are pairs of β-sheets, with the facing side chains of the two sheets interdigitated, from 13 crystal structures of such segments.

In this work, we examined 23 proteins related to amyloidoses, taken from the detailed compilations of Harrison et al. [4] and Uversky and Fink [2]. 18 of them have experimentally determined amyloidogenic regions (hereinafter called amyloidogenic determinants) [[4] and refs. therein, [17,18] and refs. therein], and of these, 7 have experimentally solved structures. The remaining 5 proteins have experimentally solved structures, but no experimentally determined amyloidogenic regions. We thoroughly examined the experimentally solved structures and the experimentally determined amyloidogenic determinants and we have found that almost all experimentally determined, and a large percentage of predicted amyloidogenic regions by our consensus prediction algorithm AMYLPRED (found at ), which predicts amyloidogenic determinants from sequence, are only partially buried into the hydrophobic cores of the solved protein structures, thus requiring only a slight (perhaps local) unfolding to occur, for the formation of aggregates and subsequent formation of amyloid fibrils.

## Results

Since proteins related to amyloidoses vary in sequence and 3D-structure and there are no profound similarities either in sequence or structure of these proteins [1,2,30], 23 such proteins were extracted from the detailed works of Harrison et al. [4] and Uversky and Fink [2] and our attempts were focused on identifying common structural features for these proteins.

18 of them have experimentally determined amyloidogenic short stretches ('amyloidogenic determinants') [[4] and refs. therein, [17,18] and refs. therein] and they are shown in Additional file 1. 7 of these 18 proteins, clearly indicated in Additional file 1, have experimentally determined 3D-structures (the relevant PDB ID's of these structures are given in this file).

The remaining 5 amyloidogenic proteins, shown in Additional file 2, have experimentally solved 3D-structures, as shown in this file, but, unfortunately, no experimentally determined amyloidogenic regions.

We developed a consensus prediction algorithm of amyloidogenic determinants, from sequence alone, called AMYLPRED (see 'Materials and Methods') and we wanted to compare its results against those of the five individual methods it combines. Therefore, we used as a test set these 23 proteins and the results are presented in detail, in Additional files 1 and 2. Table 1 presents for each method separately and for the consensus method AMYLPRED, sensitivity, specificity, the index Qα and correlation coefficient values, as these measures of accuracy were defined by Baldi at al. [32], for the 18 proteins of Additional file 1, with experimentally verified amyloidogenic regions on a per aminoacid residue basis. It also contains actual true/false positive and true/false negative values for each method to better demonstrate the bias of each individual method. On the basis of these measures, it can be seen that, AMYLPRED, performs slightly better than each individual method, as perhaps expected. In Additional file 3, the results of AMYLPRED against those of a recently developed prediction algorithm of 'hot spots' of aggregation in polypeptides, AGGRESCAN, [21] are also compared. The results of AMYLPRED on this test set, against those of two other recently presented prediction algorithms, PASTA [20] and Zyggregator [24] were also compared (data not shown).

**Table 1:** Accuracy indices of the consensus method and of its subordinate methods applied on the set of the 18 amyloidogenic proteins (see text)

*Method*	*Sensitivity*	*Specificity*	*Q*_*α*_	*Correlation coefficient*	*TP*	*TN*	*FP*	*FN*
Av. Packing Density	0.29	0.87	0.58	0.15	191	3769	575	471

SecStr	0.10	0.95	0.52	0.07	67	4107	237	595

Pattern	0.08	0.95	0.52	0.05	53	4127	217	609

TANGO	0.13	0.97	0.55	0.17	88	4207	137	574

Conf. Energy	0.39	0.79	0.59	0.14	256	3429	915	406

Consensus (3 methods)	0.13	0.95	0.54	0.11	84	4118	226	578

Consensus (2 methods)	0.31	0.88	0.59	0.18	206	3807	537	456

True/false positives (*TP*, *FP*) and true/false negatives (*TN, FN*) for each method are also shown to demonstrate better the bias of each individual method (see also text). The results for a consensus based on 3 methods are also shown.

**Figure 1:**
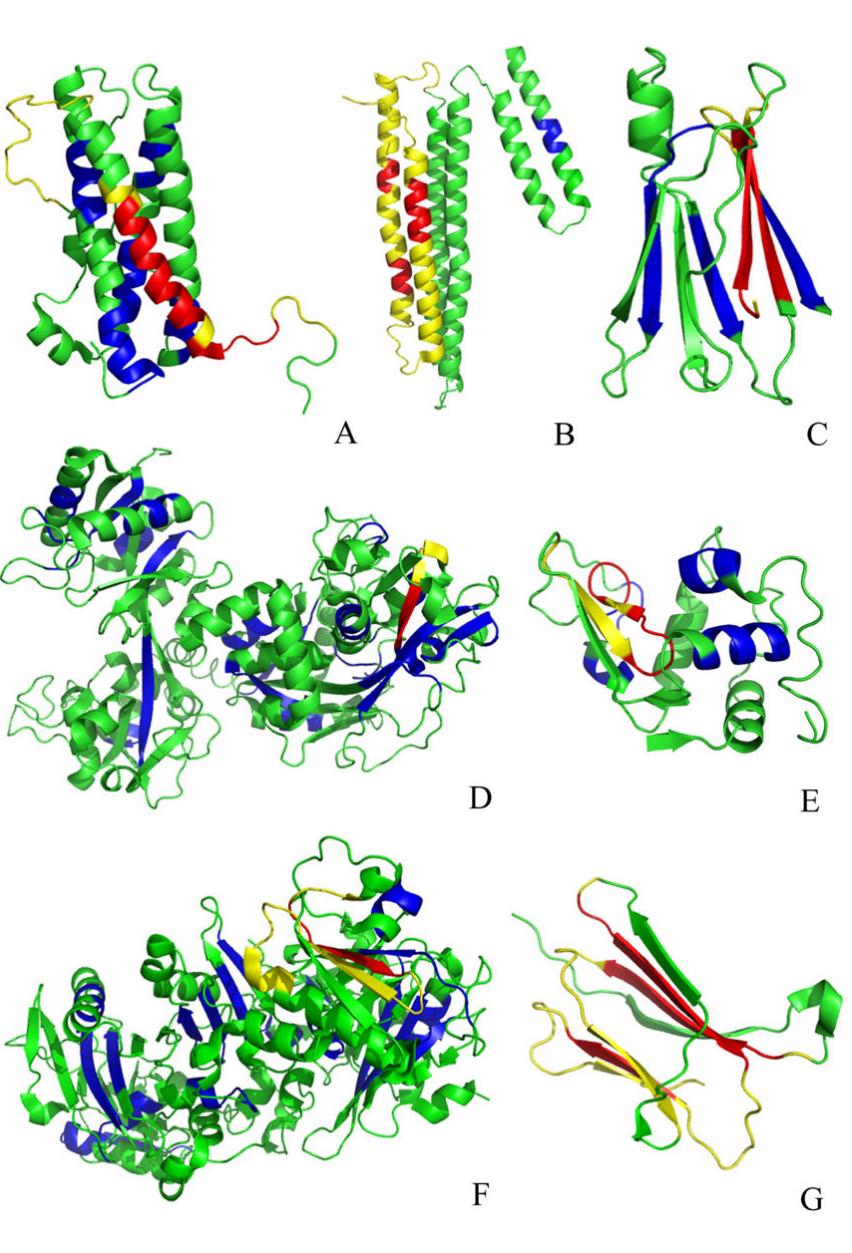
**Cartoon representations of 7 proteins related to amyloidoses, with experimentally determined structures, which contain experimentally determined amyloidogenic regions**. These 7 protein models, (see also Additional file 1), which were produced utilizing PyMOL [34] are: (A) Prolactin (PDB ID: 1RWS); (B) Apolipoprotein A-I (2A01); (C) Transthyretin (1BMZ); (D) Lactoferrin (1CB6); (E) Lysozyme C (1LZ1); (F) Gelsolin (2FGH); (G) β_2_-Microglobulin (1LDS). Experimentally determined amyloidogenic regions are shown in yellow. Theoretically predicted amyloidogenic regions, utilizing AMYLPRED (see Results), which coincide with experimentally determined regions are coloured red, whereas predicted amyloidogenic regions by AMYLPRED are shown in blue. The remainder of each protein is shown in green.

The crystal structures of 12 proteins from the set of the 23 chosen proteins related to amyloidoses are known (Additional files 1 and 2). Of these 12 proteins, 7 have experimentally determined amyloidogenic regions, shown in yellow in Figure 1, which contains cartoon representations of the determined structures. These include prolactin, apolipoprotein A-I, transthyretin, lactoferrin, lysozyme C, gelsolin and β_2_-microglobulin. Theoretically predicted amyloidogenic regions by AMYLPRED, which coincide with experimentally determined amyloidogenic determinants, are shown in red in Figure 1, whereas theoretically predicted amyloidogenic determinants by AMYLPRED, but not experimentally verified as such, are shown in blue in Figure 1.

**Figure 2:**
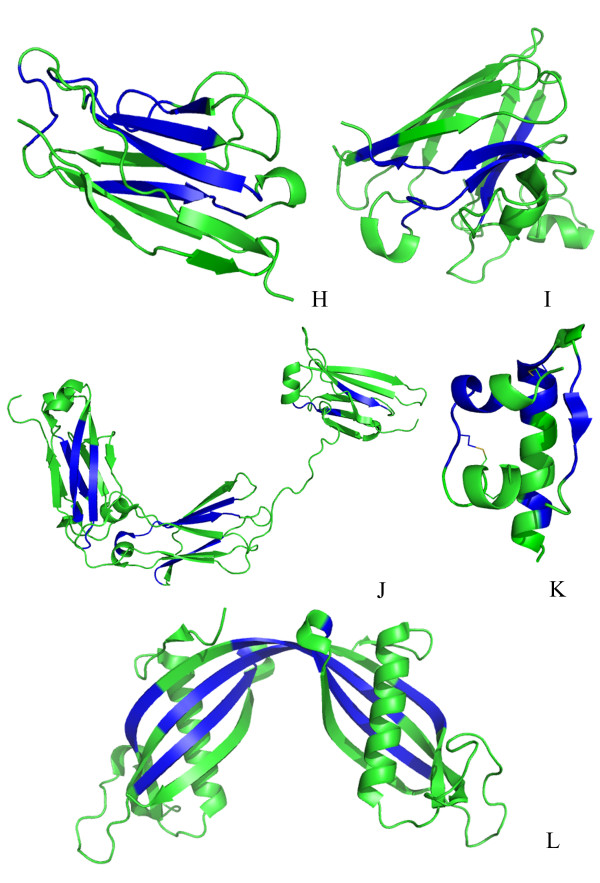
**Cartoon representations of 5 proteins related to amyloidoses, with experimentally determined structures, which do not contain experimentally determined amyloidogenic regions**. These 5 protein models, (see also Additional file 2), which were produced utilizing PyMOL [34] are: (H) Immunoglobulin κ-4 light chain (PDB ID: 1LVE); (I) Superoxide dismutase (2C9V); (J) Immunoglobulin G1 heavy chain (1HZH); (K) Insulin (1ZNJ); (L) Cystatin C (1R4C). Predicted amyloidogenic regions by AMYLPRED are shown in blue (see also Additional file 2). The remainder of each protein is shown in green.

Figure 2 contains cartoon representations of the remaining 5 protein structures, for which no experimental information for amyloidogenic determinants is currently available. These are: immunoglobulin κ-4 light chain, superoxide dismutase, immunoglobulin G1 heavy chain, insulin and cystatin C. Theoretically predicted amyloidogenic determinants by AMYLPRED on these protein structures are coloured in blue, in Figure 2.

A close examination of the 12 solved protein structures (Figures 1 and 2) by molecular graphics programs such as O [33] and PyMOL [34] and also utilizing the algorithm DSSP [35], reveals that 100% of the experimentally determined amyloidogenic determinants and ca. 70% of theoretically predicted by AMYLPRED amyloidogenic determinants, have at least one 'face' of these amyloidogenic determinants on the surface of the relevant structures, clearly not buried in the hydrophobic interiors of the protein structures. This finding was somewhat unexpected and intriguing. Furthermore, surprisingly, several of these 'not-buried' amyloidogenic determinants were predicted by the algorithm SecStr (see 'Materials and Methods') to have ambivalent propensities both for α-helix and β-sheet, in other words to have the properties of 'chameleon' sequences [36], and, also, were observed experimentally to adopt either type of secondary structure.

Quantitative estimates of total and per-residue accessible surface areas, in Å^2^, calculated using DSSP [35], for the experimentally determined amyloidogenic determinants, for the theoretically predicted by AMYLPRED amyloidogenic determinants and for other peptides, either 'exposed' on the surface or 'buried' into a protein's interior are provided in Additional file 4, for the 12 amyloidogenic proteins with experimentally determined 3D-structures of Additional files 1 &2. Additional file 5 contains quantitative estimates of total and per-residue accessible surface areas, in Å^2^, calculated using DSSP [35], for 'exposed' on the surface peptides (generally of comparable length to experimentally determined amyloidogenic determinants), taken from the structures of 9, non-amyloidogenic globular proteins, selected at random, belonging to the structural classes α, β, α + β, α/β. It can clearly be seen from these files that all experimentally found amyloidogenic determinants and a large percentage (ca. 70%) of theoretically predicted determinants, are, indeed, 'exposed' on the surface. However, it is also seen that this feature is not found only in proteins related to disease, but it is a property of globular proteins in general.

## Discussion

Amyloids are formed under protein-denaturing conditions or as a result of mutations, but they have also been observed to be the native fold of certain proteins under physiological conditions. As research continues for the understanding of the mechanisms involved in amyloid formation, the development of prediction methods is an important complement to experimental approaches.

Although, clearly, prediction tools cannot substitute experimental work, they might contribute in locating potential regions of interest for further experimental studies. Therefore, we have developed a publicly available online tool for the prediction of amyloidogenic determinants in amino acid sequences, based on the consensus of five independent prediction methods that rely on different properties of these amyloidogenic determinant-regions. In addition, we have tested the consensus method against each of its subordinate methods on the same set of 18 proteins for which experimental data is available and we have found that its results tend to be slightly more accurate than those of the individual predictors.

An intriguing finding is that signal peptides, when present (data not included in the results provided here), tend to be detected as amyloidogenic regions, usually by 4 out of the 5 methods and consequently are also shown as strong consensus hits. We currently have no explanation as to what the relation between the two features may be, besides the highly biased hydrophobic composition of the signal peptides' central region.

It is important, however, to note that the numbers shown in Table 1 are subject to change as more experimental data is acquired, because regions currently marked as non-amyloidogenic are not necessarily so and may prove to be in fact amyloidogenic in the future. Indeed, several regions were found in these 18 proteins, for which there was a strong agreement among all methods but are currently marked as non-amyloidogenic. Such prediction results may suggest amyloidogenic determinants currently unknown and methods like the one presented here might therefore provide valuable hints to experimental researchers. It should perhaps be mentioned at this point that, recently, we synthesized and structurally studied six (6) peptides from different proteins of Additional file 1, that the AMYLPRED tool predicts as amyloidogenic determinants (not previously experimentally verified as such) and we have found that five (5) of them produce amyloid fibrils, in water, at physiological pH, temperature and ionic strength, apparently having the ability to act as amyloidogenic determinants (Iconomidou & Hamodrakas, In preparation). This, most probably indicates that AMYLPRED might be a useful tool to experimental researchers. However, it should perhaps be emphasized that the use of AMYLPRED does not provide insights on the molecular rules underlying the aggregation event, as other tools, like TANGO [13], actually do.

Of course, it may be argued that most false positives, which result in the low correlation values presented in Table 1, are mainly due to consensual overpredictions, since all currently available amyloid prediction methods are notorious for their high degree of overprediction, and this perhaps is further seen in Additional file 3, where the results of AMYLPRED are compared with those derived by a recently developed method AGGRESCAN [21] and also when compared against those of two other recently presented prediction algorithms PASTA [20] and Zyggregator [24] (data not shown). However, it is obvious that further experiments are needed, which may reveal important clues for the amyloidogenic properties of the relevant proteins.

Also, it should be said that questions may be raised about the statistics provided in Table 1, as well as the data set used to generate these statistics: ideally the data set should be composed of more or less equal amounts of experimentally verified positives and negatives, allowing to score both false negatives and false positives. As the experimentally verified set is only composed of positives, only false negatives can be scored.

Nevertheless, in this study, we demonstrated rather conclusively that, practically all experimentally determined amyloidogenic determinants, to date, and more than 70% of predicted, but not yet experimentally verified short potential amyloidogenic stretches, are placed on the surface of the amyloidogenic proteins (see 'Results'). Furthermore, several of them have the ability to act as conformational 'switches' (see 'Results'). This may signify that aggregation and amyloid formation is mediated via such short stretches, which may be achieved by partial local unfolding. It is perhaps difficult to reconcile this observation with the hypothesis that protein unfolding should occur prior to aggregation, however, these short stretches may act as 'switches', for partial unfolding of the whole protein. Experiments, focused on these fragments, should be performed to test this idea. In this respect, it interesting to note that, the peptide VEALYL, which appears in the crystal structure of insulin (PDB ID 1ZNJ, chain B, residues 12–17) adopts an α-helical conformation, with Leu(15) buried into the hydrophobic interior of the insulin monomer (chains A and B), whereas, when crystallized alone, forms a steric zipper, class 7 (see [31]), adopting an extended β-strand conformation. At the same time, AMYLPRED predicts it as an amyloidogenic determinant (Additional file 2), with its subordinate program SecStr classifying it as a 'chameleon' sequence, that is, a sequence with ambivalent propensity both for α-helix and β-sheet. This observation may have important implications for the amyloidosis related to insulin, namely iatrogenic amyloidosis [4], presumably by finding factors that may stabilize the conformation of this peptide as α-helical, *in vivo*. Thinking along similar lines may lead to a number of interesting practical consequences for other amyloidoses related to proteins of known 3D-structure, with experimentally verified amyloidogenic determinants, accessible on the surface of the proteins (Figure 1 and Additional file 1).

## Conclusion

The results of this study clearly suggest that nearly all experimentally determined amyloidogenic determinants and a large percentage of predicted, but not yet experimentally verified short potential amyloidogenic stretches, are found on the surface of the relevant proteins, 'exposed' to the surrounding solvent and to interactions with neighbouring molecules. Furthermore, several of them have the ability to act as conformational 'switches', for partial unfolding of the whole protein. Experiments, focused on these fragments, should be performed to test this idea.

## Methods

### Tools and databases used

Amino acid sequences of the proteins used in this study were retrieved from UniprotKB [[37], ]. Protein structures were retrieved from PDB [[38], ]. For each protein structure used, residues accessible to the solvent or buried into a protein's hydrophobic interior were determined utilizing the algorithm DSSP [35] and checked by visual inspection of the relevant structures utilizing the molecular graphics programs PyMOL [[34], ) and O [33]. Cartoon drawings of the structures were obtained using PyMOL [34], ).

### The consensus prediction tool AMYLPRED

For the purpose of this work, to produce a web-tool that would perform a consensus prediction of amyloidogenic determinants from protein sequences, utilizing available algorithms, we have used five different methods whose algorithms are publicly available or readily implementable and whose input is protein primary structure data.

The first method relies on average packing density profiles [17,18]. No algorithm has been published for this method, therefore we implemented our own.

The second method used is the online consensus secondary structure prediction algorithm SecStr [26] that has been shown to be able to predict amyloidogenic regions as conformational switches [25], which are identified as regions predicted both as α-helices and β-strands. SecStr [26], predicts separately α-helices and beta-strands. Regions predicted both as α-helices and beta-strands, by three individual methods of SecStr at least, are considered as conformational switches (chameleon sequences) [25]. These are easily identified, inspecting the text ouput file of SecStr .

Locating the amyloidogenic pattern {P}-{PKRHW}-[VLSCWFNQE]-[ILTYWFNE]-[FIY]-{PKRH} [11] is another method used for our consensus prediction and is carried out by a short custom-written script.

The TANGO algorithm [13] is the next method used (version 2.1). It calculates the tendency of peptides to form beta aggregates and aside from the primary sequence, it also requires a set of environmental variables to be set. As a universal approach applicable to all proteins, the default values for these variables from the TANGO web-server submission page have been chosen.

Finally, an algorithm that maps all hexapaptides of a sequence onto the microcrystalline structure of NNQQNY and calculates the resulting conformational energy is also used [22]. Minor modifications to the source code of this algorithm have been made in order to allow for its automated execution.

The consensus prediction was found to produce the best results when the threshold is set to require overlapping hits by at least two of the five methods used. The consensus prediction is presented in the web browser window, while the complete predictions by all methods are made available as a downloadable text file. The consensus prediction tool is freely available to academic users at: . However, non-academic users of this algorithm should obtain permission of its use from the authors of the original algorithms and the corresponding author of this article.

### Amyloidogenic protein data sets

23 proteins related to amyloidoses, were taken from the detailed compilations of Harrison et al. [4] and Uversky and Fink [2]. 18 of them have experimentally determined amyloidogenic regions (hereinafter called amyloidogenic determinants) [[4] and refs. therein, [17] and refs. therein] and of these 18 proteins, 7 have experimentally solved structures. The remaining 5 proteins have experimentally solved structures, but no experimentally determined amyloidogenic regions yet. All proteins were cleared of signal peptides, pro-peptides and other chains that are present in their database entries in UniprotKB [37] but are not part of the mature protein, and the exact locations of the experimental regions were identified by referring to the respective original publications [[17,18] and references therein, [4] and references therein].

Here, it should perhaps be mentioned that, the list of the 23 proteins contains also proteins that their structure is known either for fragments or in conditions that is not at all certain that they are similar to the conditions *in vivo*. Such proteins are calcitonin (for example PDB ID 2GLH, in sodium dodecyl sulfate micelles), major prion protein (the structure of several fragments of the prion protein is known), IAPP (for example 1KUW in detergent micelles or 2F48-human insulin-degrading enzyme in complex with IAPP), α-synuclein (for example 1XQ8, micelle bound α-synuclein). Although the results of this study apply to these proteins too, they were not taken into account.

### Specific points concerning the quality of the Amyloidogenic protein data sets and application of AMYLPRED and of its subordinate methods

There is an average of less than 1.5 known amyloidogenic regions per protein in the dataset, while the consensus method offers an average of 5 predicted stretches per protein and similar if not even more over-predictive results are given by the methods of Galzitskaya et al. [17,18] and Zang et al. [22]. The total dataset collectively amounts to 5006 aminoacid residues and the total amount of experimentally positive residues is only 662, which represents 13% of the dataset. The set is thus highly biased by negatives. This imbalance is responsible for the low correlation values (see 'Results'). However we chose this data set, not as a test set of the performance of the consensus algorithm, AMYLPRED, but as a representative set of well known proteins related to amyloidoses, with well defined amyloidogenic properties, structure and experimentally known determinants (if possible), in our attempts to find common features of predicted, or experimentally known amyloidogenic determinants.

Results are judged based on the correlation coefficient values in order to offer a better overall prediction. Concensus is based on 2 methods instead of 3, because a concensus based on 3 methods produces a considerably lower correlation coefficient value (0.11 instead of 0.18) as a result of positive results being greatly reduced and lowering the sensitivity from 0.31 to 0.13 (see Table 1).

As it is apparent from the 'Results' section, the performance of the consensus method, AMYLPRED, is not spectacularly better than that of the individual methods. This is likely because combining the strengths of the algorithms without mixing their weak points is not a task that can be attained with a linear combination of the methods.

This is better seen when all algorithms and AMYLPRED were applied to a relatively well-balanced data set of 179 peptides used by Serrano and co-workers [[13], Additional file 1] to test the TANGO algorithm performance [13]. This data set contains 66 peptides, experimentally found to aggregate and 113 peptides also known not to aggregate by experiment. The results are shown in Additional file 6. The consensus algorithm, AMYLPRED (correlation coefficient, C = 0.58) performs slightly worse than the Conformational energy [22] algorithm (C = 0.65) and equally well to TANGO [13] (C = 0.58) with default parameters that was used by us in this work. However, they all perform worse than TANGO (C = 0.75), with the environmental variables set by its authors [13]. It should perhaps be mentioned at this point that, the correlation coefficient values were calculated on a per segment basis rather than a per residue basis in this case.

There has been consideration for an implementation of a weighted contribution of the methods, possibly performed by a neural network. The gain from such an endeavor however would probably not be very great and would be overshadowed by the inherent flaws of all methods.

However, the main result of AMYLPRED is not the list of consensus hits. It is the agreement profile of the methods (graph in the text output file) that provides a better insight on the results. The consensus hits shown on the webpage are presented as a convenient output for quick scans, but they lack the crucial information of how strong a prediction actually is, which is perhaps the most important factor in choosing regions for potential experimental research. When applying AMYLPRED, care should be taken that it requires a minimal overlap of 2 residues at least, in order to indicate a positive result.

## Authors' contributions

KKF developed the web-tool, analyzed the data, and drafted the manuscript. VAI participated in the coordination of the study, re-analyzed and checked the data and helped editing the manuscript. CMK retrieved the protein data set, examined the solved structures, calculated the accessibility of the peptides and prepared the protein cartoons. SJH designed and co-ordinated the study and was responsible for overall supervision. All authors read and approved the final manuscript.

## Supplementary Material

Additional file 1 **Prediction of amyloidogenic determinants, for 18 proteins, with experimentally verified amyloidogenic regions, in the evaluation set, for each method separately and for the consensus method (AMYLPRED)**. Details are given in the file. Click here for file

Additional file 2 **Prediction of amyloidogenic determinants, for 5 proteins, without experimentally verified amyloidogenic regions, for each method separately and for the consensus method (AMYLPRED)**. Details are given in the file. Click here for file

Additional file 3 **Prediction of amyloidogenic determinants, for the 23 proteins of Additional files **1 &2, **both by AMYLPRED and AGGRESCAN (Conchillo-Solé et al., 2007), for comparison**. Details are given in the file. Click here for file

Additional file 4 **Quantitative estimates of total and per-residue accessible surface areas, in Å^2^, calculated using DSSP **[35], **for the experimentally determined amyloidogenic determinants, for the theoretically predicted by AMYLPRED amyloidogenic determinants and for other peptides, either 'exposed' to the solvent or 'buried', for the 12 amyloidogenic proteins with experimentally determined 3D-structures of Additional files **1 &2. Details are given in the file. Click here for file

Additional file 5 **Quantitative estimates of total and per-residue accessible surface areas, in Å^2^, calculated using DSSP **[35], **for 'exposed' to the surface peptides (generally of comparable length with those of experimentally determined amyloidogenic determinants), taken from the structures of 9 non-amyloidogenic globular proteins, belonging to the structural classes: alpha, beta, alpha+beta, alpha/beta**. Details are given in the file. Click here for file

Additional file 6 **Accuracy indices of the consensus method, AmylPred, and of its subordinate methods, applied on a balanced set of 179 peptides, used by Serrano and co-workers to test the TANGO algorithm (see text)**. True/false positives (*TP*, *FP*) and true/false negatives (*TN*, *FN*) for each method are also shown to demonstrate better the bias of each individual method (see also text). The results for a consensus, AmylPred, based on 2 methods are also shown. TANGO* is the algorithm we used, with default parameters, whereas, TANGO** is the algorithm used by Serrano and co-workers, with the environmental variables set by its authors [13]. Correlation coefficient values were calculated on a per segment, rather than a per residue basis. Click here for file
