# Supplementary material for: Amyloidogenic determinants are usually not buried
Source: BMC Struct Biol. 2009 Jul 9;9:44. doi: 10.1186/1472-6807-9-44 (PMC2714319; doi:10.1186/1472-6807-9-44)
Supplement: Additional file 6 — Accuracy indices of the consensus method, AmylPred, and of its subordinate methods, applied on a balanced set of 179 peptides, used by Serrano and co-workers to test the TANGO algorithm (see text). True/false positives (TP, FP) and true/false negatives (TN, FN) for each method are also shown to demonstrate better the bias of each individual method (see also text). The results for a consensus, AmylPred, based on 2 methods are also shown. TANGO* is the algorithm we used, with default parameters, whereas, TANGO** is the algorithm used by Serrano and co-workers, with the environmental variables set by its authors [13]. Correlation coefficient values were calculated on a per segment, rather than a per residue basis. [file 1472-6807-9-44-S6.doc]

**Additional File 6**

Accuracy indices of the consensus method, AmylPred (with bold), and of its subordinate methods, applied on a balanced set of 179 peptides, used by Serrano and co-workers to test the TANGO algorithm (see text). True/false positives (*TP*, *FP*) and true/false negatives (*TN, FN*) for each method are also shown to demonstrate better the bias of each individual method (see also text). The results for a consensus, AmylPred, based on 2 methods are also shown. TANGO* is the algorithm we used, with default parameters, whereas, TANGO** is the algorithm used by Serrano and co-workers, with the environmental variables set by its authors [13].

| *Method* | *Sensitivity* | *Specificity* | *Q_α_* | *Correlation coefficient* | *TP* | *TN* | *FP* | *FN* |
| --- | --- | --- | --- | --- | --- | --- | --- | --- |
| Av. Packing Density | 0.65 | 0.82 | 0.74 | 0.48 | 43 | 93 | 20 | 23 |
| SecStr | 0.44 | 0.88 | 0.66 | 0.36 | 29 | 99 | 14 | 37 |
| Pattern | 0.42 | 0.93 | 0.68 | 0.43 | 28 | 105 | 8 | 38 |
| TANGO* | 0.61 | 0.93 | 0.77 | 0.58 | 40 | 105 | 8 | 26 |
| Conf. Energy | 0.88 | 0.79 | 0.83 | 0.65 | 58 | 89 | 24 | 8 |
| **AmylPred** | **0.74** | **0.84** | **0.79** | **0.58** | **49** | **95** | **18** | **17** |
| TANGO** | 0.82 | 0.92 | 0.87 | 0.75 | 54 | 104 | 9 | 12 |
